# Daily Negative Work Events and Employees' Physiological and Psychological Reactions

**DOI:** 10.3389/fpsyg.2016.01711

**Published:** 2016-11-08

**Authors:** Judith Volmer, Andrea Fritsche

**Affiliations:** ^1^Work and Organizational Psychology Group, Department of Psychology, University of BambergBamberg, Germany; ^2^Social Psychology Group, Department of Psychology, University of ErlangenErlangen, Germany

**Keywords:** allostatic load model, negative work events, cortisol activity, negative affect, emotional exhaustion

## Abstract

Scholars have accumulated an abundant amount of knowledge on the association between work stressors and employees' health and well-being. However, notions of the complex interplay of physiological and psychological components of stress reactions are still in their infancy. Building on the Allostatic Load (AL) model, the present study considers short-term within-person effects of negative work events (NWEs) on indicators of both physiological (i.e., salivary cortisol) and psychological distress responses (i.e., negative affect and emotional exhaustion). Multilevel findings from an experience sampling study with 83 healthcare professionals suggest that reported NWEs predict employees' psychological but not endocrine stress responses. Results contribute to a more comprehensive understanding of employees' daily response patterns to occupational stressors.

## Introduction

An important goal of organizational researchers is to identify effective ways to foster workers' health and well-being. At work, employees often encounter stressful events, such as conflicts with customers or interruptions that hinder goal achievement. Research has acknowledged the importance of stressful situational demands for employee well-being (for a review, see Sonnentag, 2015). However, the impact of negative work events (NWEs) on physiological distress remains scarcely investigated (for exceptions, see Ilies et al., 2010a; Stawski et al., 2013). In addition, findings are inconsistent: While some studies provide evidence for an increase in cortisol secretion in response to stressful events (van Eck et al., 1996; Wirtz et al., 2013), others yield opposite results (Manuck et al., 1991; Hjortskov et al., 2004). Further, most studies that have considered physiological outcomes employed laboratory or experimental designs (for a meta-analytical discussion, see Dickerson and Kemeny, 2004), resulting in low ecological validity. Most importantly, a direct comparison of psychological and physiological well-being reactions to daily NWEs has not been drawn so far (for an exception, see Vedhara et al., 2003). This is surprising, as the examination of both physiological and psychological well-being indicators is indispensable to gain a more complete picture of employees' reaction patterns to taxing occupational demands.

We aim at extending previous research on daily response patterns to occupational stressors in the following ways: First, we use an event-related fixed-occasion design (Kudielka et al., 2012) to be able to link daily measurements indicative of physiological and psychological distress directly to the respective NWEs. Thereby, we take account of recent calls to apply ecological momentary assessement (EMA) in work and occupational health psychology (Ilies et al., 2016). Second, we compare within-person effects of NWEs on both physiological and psychological well-being indicators. As a biomarker, we include cortisol, as it has been identified as an integral neuroendocrinological component of stress responses (Sapolsky, 2002). Regarding psychological well-being, we include negative affect and emotional exhaustion. State negative affect, characterized by feelings of anxiety, worry, sadness, or unease, constitutes an appropriate indicator of distress (Watson et al., 1988). Likewise, emotional exhaustion as the core dimension of burnout has been established as a useful stress indicator (Reichl et al., 2014). Third, our study builds on the allostatic load model of stress (AL Model; McEwen and Stellar, 1993; McEwen, 2007) as an organizing theoretical framework.

## The body's stress-response system

The human body is capable of reacting efficiently to short-term physical stress to bring the organism back to its original condition (Sapolsky, 2002). Stress responses require high amounts of energy, so that physiological systems are energized in order to deal with a threat (Kemeny, 2003). At the same time, bodily functions that are not involved in the quick mobilization of energy, such as digestion, reproductive behavior, inflammation, or pain perception, are suppressed. Yet, when an organism is regularly exposed to chronic stressors or when the stress response is evoked by psychological rather than by physiological stressors, stress-related diseases may develop (Sapolsky, 2002; Ganster and Rosen, 2013).

### Stress and HPA axis regulation

One of the major stress response systems in the body is the hypothalamic-pituitary-adrenal (HPA) axis and its “end-product” cortisol (Kudielka et al., 2012). The HPA axis comprises the hypothalamus, the pituitary, and the adrenal cortex. Nerves that are confronted with a stressor get stimulated and cause the hypothalamus to secrete corticotropin-releasing hormones. This in turn leads to the secretion of adrenocorticotropic hormones from the pituitary which consequently provokes the release of the steroid hormone cortisol (also called hydrocortisone) from the adrenal cortex.

Cortisol triggers metabolic processes in the body that provide energy. This is the reason why cortisol is a crucial part of the stress-response, as the immediate mobilization of energy is one of its most important features (Sapolsky, 2002). As a biomarker of psychological stress and related mental or physical diseases, cortisol is routinely used (Hellhammer et al., 2009).

### Cortisol characteristics

Cortisol, like most other hormones, follows a specific circadian pattern or rhythm (Gorman and Lee, 2002). Cortisol secretion shows peak levels shortly after awakening in the morning. This phenomenon is described as the so-called “cortisol awakening response” (Kudielka et al., 2012). During the rest of the day, cortisol levels decrease continuously.

It is recommended to measure cortisol samples several times a day. First, it is important to avoid confounding cortisol reactions to stressful events with regular changes in the diurnal cortisol secretion. Second it is necessary to ensure that all short-term cortisol reactions to stressful events are recorded. Different indicators of the cortisol daily profile or cortisol output have been used in assessments, such as the difference between waking and bedtime cortisol levels or the total output measured by the area under the curve (AUC). To assess total daily output, a curve is created using several cortisol samples from different times of the day. Then, the area below this curve is measured (Pruessner et al., 2003).

## Allostatic load model

A model that describes an organism's reaction to stressful experiences in various stages, starting from an initial stress response to the disease endpoint, is the Allostatic Load (AL) model (Ganster and Rosen, 2013). This model replaces the long-lasting belief that organisms try to maintain a state of homeostasis to keep their internal environment stable in reaction to stress with the concept of allostasis, which in turn focuses on “the ability to achieve stability through change” (McEwen, 1998, p. 171).

The AL model specifies three levels representing different adaption systems (i.e., primary, secondary, and tertiatry processes) which interact with each other. The perception of a potential environmental stressor triggers an initial adaption in the central nervous system which in turn manifests in psychological, psychosomatic, and physiological processes. Stress hormones, such as cortisol, are stimulated as well and can be understood as primary mediators. Primary processes prepare the organism to cope with threats to its balance. This process functions very well if the stressor is short-termed and not of psychological nature, but if the stressor is repeatedly present, primary mediators are chronically activated, and allostatic load occurs.

Constant activation of primary processes of initial adaption triggers secondary processes of allostatic load which imply that various biological systems alter their normal operating ranges (set points). These include the immune, cardiovascular, and metabolic systems. During the body's response to stress, these systems show a different functionality to which they become accustomed if the stressor persists for a longer period of time. For example, the immune system is suppressed and only little resources are available in order to produce antibodies. After a while, the system gets used to this low production of antibodies and changes its set point. From then on, fewer antibodies will be produced, even if stressors are absent. The longer secondary perturbations exist, the more likely the organism will reach the tertiary phase of allostatic overload, which represents disease endpoints (e.g., cardiovascular disease), psychological disorders (e.g., clinical depression), or all-cause mortality. Primary and secondary mediators are important predictors for tertiary health outcomes (e.g., cardivascular disease, diabetes, depression, and even death). While secondary mediators are relatively easy to sample (e.g., blood pressure), the sampling of primary mediators, such as cortisol, is more challenging.

The AL model can be applied in a work context (cf. also Ilies et al., 2016). Confronted with negative events at work or stressors, a person will show psychological (e.g., increased negative affect and exhaustion) and physiological (e.g., increased cortisol secretion) reactions. For instance, primary mediators, such as cortisol, will be produced in higher quantities and when the stressors fade, the cortisol level should sink to its original condition. If the stressor persists for a longer period of time, as can be the case with many work-related problems, or if several stressors occur quickly one after another, primary mediators will rise to a level where the organism is no longer capable of changing its set point and so secondary mediators like HPA axis dysregulation occurs, which can then lead to adverse tertiary health outcomes. Constantly high cortisol levels or very high cortisol reactions to stressors should therefore be predictors of work related illnesses resulting from stress. Yet, findings on the relationship between NWEs and resulting cortisol levels have not been completely consistent. Furthermore, a direct comparison of psychological and physiological distress reactions to daily NWEs is still missing.

## Development of hypotheses

### Associations between NWEs and physiological well-being

Dickerson and Kemeny (2004) conducted a meta-analysis of 208 studies on plasma and salivary cortisol responses to stressors. They found that, while stressors generally raised cortisol levels, effects varied strongly between tasks. In a more recent field study, Stawski et al. (2013) examined links between the occurrence of daily stressors and the secretion of salivary cortisol in a sample of 1694 adults over four consecutive days. Their core assumption was that daily stressors would increase diurnal cortisol output (AUC). Stawski et al. (2013) collected saliva immediately after waking up, 30 min after waking-up, before lunch, and at bedtime. Information about daily stressors was assessed retrospectively with telephone interviews. Findings suggest that within-persons AUC as well as cortisol levels after waking up and before lunch were significantly higher on days with many stressors compared to stressor-free days. However, events reported in interviews after having finished work are likely to be affected by retrospective bias. Moreover, direct effects of NWEs on salivary cortisol remained uninspected, as salivary sampling was not attached to specific events but delivered on fixed occasions. Examining the occurrence of stressful events in naturalistic settings, such as the workplace, should lead to a similar outcome as presented in various laboratory studies (Dickerson and Kemeny, 2004). Thus, the first two hypotheses to test the effects of daily NWEs on employees' physiological well-being are:

***Hypothesis 1***. On days employees report more NWEs their AUC will be more elevated than on days with fewer reported NWEs.
***Hypothesis 2***. On days employees report more NWEs their cortisol levels after finishing work will be higher than on days with fewer NWEs.

### Associations between NWEs and psychological well-being

People are strongly affected by NWEs, as these signals that one is not on the “right track” and that one has to adjust one's behavior. A widely investigated distress indicator when it comes to short-effects of workplace stressors is negative affect. People spend more time reasoning about negative experiences compared to positive experiences (Abele, 1985). As people's long-term memory is influenced by the affective states when they encode the respective information (Bower, 1981), NA should increase the likelihood of retrievals of NWEs right after finishing work. Empirically, Ilies et al. (2011) showed that daily interpersonal conflicts were positively associated with negative affect after work in a sample of 49 university employees. Volmer et al. (2012) demonstrated that daily customer conflicts predicted negative affect at bedtime in a sample of 98 civil service agents. We therefore assume that daily NWEs are positively associated with negative affect right after finishing work.

NWEs distract attention from task completion and are highly time- and effort-consuming. Consequently, when individuals experience unpleasant customer interactions or conflicts with colleagues and/or supervisors, resources are likely to be depleted (Hobfoll and Freedy, 1993; Brotheridge and Grandey, 2002) because task and social goals have not been accomplished. Consequently, regarding emotional exhaustion as a primary burnout indicator, research has shown that NWEs are positively associated with emotional exhaustion (Grandey et al., 2004) through cognitive rumination (Baranik et al., in press). We therefore assume that daily NWEs are positively associated with emotional exhaustion right after finishing work. In sum, we hypothesize:

***Hypothesis 3***. On days employees report more NWEs their level of negative affect right after finishing work is higher than on days with fewer NWEs.
***Hypothesis 4***. On days employees report more NWEs their level of emotional exhaustion right after finishing work will be higher than on days with fewer NWEs.

## Methods

### Sample

Participants were recruited via flyers and intranet postings and included 83 German clinical staff members (62.7% females). Mean age was 40.76 years (*SD* = 11.84) with an average of 11.31 years (*SD* = 9.00) of job tenure. The majority of participants underwent professional training (55.29%), followed by participants who received higher professional training (21.18%), and participants who had a university degree (20.00%). Mean body mass index (BMI) was 25.53 (*SD* = 4.41) and 33.7% of the participants were smokers. To raise compliance, an incentive of 50€ was given if the participant provided the necessary data on all of the 3 days of the study. If the participant only delivered data for 1 day, he or she received only 15€ and for 2 days 30€, respectively.

### Procedure

One week in advance of the diary study, participants completed a questionnaire to provide sociodemographic information and control variables (e.g., height, weight, medication). A combinated time- and event-contingent event-sampling approach was used to assess daily NWEs and employees' physiological (i.e., salivary cortisol) as well as psychological (i.e., negative affect right after finishing work and emotional exhaustion right after finishing work) stress levels. Participants were instructed to report NWEs 1 h after starting work and right after finishing work (time-contingent; cf. Table 1 for details). Solely NWEs that had occurred within the last 30 min had to be reported. It was pointed out that both major and minor events should be indicated, but only events related to work. Events could also be reported between the two fixed occasions if participants had experienced NWEs in the meantime (event-contingent). Thus, there were two optional non-fixed occasions.

**Table 1:** **Collection times and descriptive statistics for non-transformed salivary cortisol levels**.

**Sample collection times^a^**	***M* in hours**	***SD* (BP) in minutes**	***SD* (WP) in minutes**
Waking cortisol	06:12	92	44
1 h after starting work	09:37	129	94
First non-fixed occasion	13:12	150	109
Second non-fixed occasion	13:48	175	86
Right after finishing work	16:22	181	116
**Cortisol samples (nmol/l)**	***M***	***SD*** **(BP)**	***SD*** **(WP)**
Waking cortisol	12.58	6.05	5.16
1 h after starting work	7.75	4.43	2.64
First non-fixed occasion	6.52	5.25	2.62
Second non-fixed occasion	5.54	3.95	1.81
Right after finishing work	3.99	2.67	1.49
AUC	73.47	32.77	17.54

*SD (BP), standard deviation between-persons; SD (WP), standard deviation within-person*.

a *As measured by the electronic track cap*.

Salivary cortisol samples were provided after waking up in the morning, 1 h after starting work, right after finishing work, and every time an event was reported in between the two fixed occasions. HPA axis reaction reaches its peak 15–40 min after the experience of a stressful event (Dickerson and Kemeny, 2004; Schlotz et al., 2008). Hence, the instruction to only report events that had occurred during the last 30 min.

To ensure compliance, the cotton-swabs that had to be used for salivary sampling were stored in a container closed with an electronic track cap (so called MEMSCap; MWV Aardex, Switzerland) that would save the exact time and date each time the container was opened. On each booklet, participants had to write down the date. On every occasion they gave a salivary sample, they had to write down the exact time and date. Participants had been told about the time-tracking caps and the importance of providing exact information about the times of sampling. These measures have been taken because other studies using salivary cortisol samples had shown that adherence to sample times was quite low (e.g., Halpern et al., 2012) and because non-compliance to sampling times can bias resulting cortisol data (Kudielka et al., 2012). Bivariate correlations between time as written down by the participants and as measured by the track cap were very high (*r*s > 0.98, *p*s < 0.001), indicating that the measures taken to ensure compliance in this study were successful.

### Measures

Event-sampling was used to assess *daily NWEs* (cf. Gross et al., 2011). On each occasion (two fixed occasions: 1 h after having started work and right after finishing work; two non-fixed occasions), participants were asked to describe a negative event that had occurred during the last 30 min. We included both, the number of fixed and non-fixed occasions NWEs into our analyses.

### Physiological distress reactions

To measure *cortisol*, participants received a container with cotton-swabs (*Salivettes*; Sarstedt, Germany). Each time they gave a salivary sample, subjects took a cotton-swab, chewed on it for about 2 min, and subsequently put it in a plastic tube on which date and time of the sample were registered. Participants were advised not to eat, drink, smoke or brush their teeth within 30 min before giving a saliva sample, so as not to distort salivary cortisol levels (Kudielka et al., 2012). Saliva samples were stored at −60°C. By means of radioimmunoassay (RIA), cortisol concentrations were analyzed in an endocrinological laboratory at a large German university. Overall RIA quality was in accordance with established RIA standards (Liening et al., 2010).

We calculated the AUC as well as cortisol levels after finishing work. Pruessner et al. (2003) provide a detailed guide on how to calculate the AUC. To better understand what the AUC is, one can imagine a coordinate system with the number of measurements as x-coordinate and the cortisol levels as y-coordinate. Values of a subject's cortisol levels at different time points are then entered in the coordinate system and connected to a graph. The area below the graph is the so-called area under the curve. This area gets divided in triangles and rectangles which can be measured and then added together to result in the AUC.

### Psychological distress reactions

Following Sonnentag et al. (2008), we assessed *daily negative affect* at the end of the workday with six items from the Positive and Negative Affect Scale (PANAS; Watson et al., 1988). On 5-point Likert scales (1 = *not at all*, 5 = *very much*), employees rated the intensity of their momentary affective experience of emotional states described by adjectives such as *nervous, distressed*, and *scared*. Cronbach's alpha ranged from 0.80 to 0.86 over the 3 days (mean α = 0.82).

*Daily emotional exhaustion* was measured at the end of the workday with seven items from the Oldenburg Burnout Inventory (OLBI; Demerouti et al., 2003). Participants indicated their agreement on 4-point Likert-scales (1 = *totally disagree*, 4 = *totally agree*). A sample item was “Today, I felt emotionally exhausted at work.” Cronbach's alpha ranged from 0.83 to 0.88 over the 3 days (mean α = 0.86).

### Data analytic approach

Because our data had a hierarchical structure, where measurements were nested within individuals, we employed hierarchical linear modeling in HLM (Version 6.08; Raudenbush et al., 2009). Multilevel models were estimated by means of full maximum likelihood. Day-level predictor variables were centered at the person mean, while control variables were centered at the sample mean to facilitate a meaningful interpretation of estimates.

### Data reduction

Compliance was assessed by comparing the time points indicated by the participants on the questionnaires and the time points measured by the electronic track caps. Thus, in cases where time designations differed by more than an hour, cortisol values of the corresponding measurement occasions were excluded from further analyses. Moreover, time points of reported NWEs, as indicated by the participant, were compared with the time points of the saliva sample, as measured by the track cap. On average, cortisol rises ~20–40 min after exposure to a stressor and returns to its baseline level ~40–60 min after the occurrence of the stressor (Dickerson and Kemeny, 2004). For this reason, measuring cortisol as a reaction to a stressor that has occurred longer than an hour ago does not provide useful information. If the time lag between a reported NWE and a saliva sample was longer than an hour, the respective cortisol value was excluded from further analyses. In total, 15 cortisol values were removed during the compliance check.

### Cortisol analyses

To turn the viscous and sticky consistency of the saliva samples more watery, salivary samples were thawed and centrifuged three times to break long molecule-chains (Schultheiss et al., 2012). In this state, the saliva samples would be easier to pipette. To speed up the thawing, warm water baths were used. Afterwards, the samples were spun for 10 min at 1000 g in a centrifuge, so that the coarse content in saliva was pushed to the ground of the tube. The watery part was pipetted into a new tube and the centrifugation procedure was repeated once more in order to eliminate all coarse content left in the saliva. In this state, the salivary samples were ready to be analyzed in a radioimmunoassay (RIA).

Cortisol levels were determined by solid-phase 125I radioimmunoassays (Coat-A-Count; Diagnostic Products Corp., Los Angeles, CA). The main assay quality parameters are sensitivity, accuracy, and precision. Schultheiss and Stanton (2009) give detailed instructions on how to calculate these parameters. Sensitivity is defined as the lowest dose of a hormone analyte that can be differentiated from a sample with no analyte. For this study, 12 RIAs were conducted and the mean analytical sensitivity was 0.14 ng/ml (*SD* = 0.02). In nmol/l the mean was 0.38 nmol/l (*SD* = 0.04).

The accuracy parameter depicts the ability of the assay to measure the true concentration of the hormone in the sample being tested (Schultheiss and Stanton, 2009). It was measured by including control samples with known amounts of cortisol in the assay and then comparing the estimated amount of the hormone by the assay with the actual amount added (in this study 1.5 and 3.5 ng/ml cortisol). The result was the percentage of the actual amount that was estimated by the assay, the so-called recovery coefficient (RC). The RCs of the 12 RIAs of this study ranged from 94.20 to 99.79% which reflects good accuracy.

The precision parameter depicts the level of agreement between test results that are repeatedly and independently obtained under stable conditions (Schultheiss and Stanton, 2009). The mean intra-assay CV of all 12 RIAs run was 5.02%. Generally, intra-assay CVs below 10% are considered as good. All RIAs met this standard, except for one RIA that showed an intra-assay CV of 24.65%. This is an unusually high CV which may be due to inaccurate pipetting, bad curve fitting or insufficient pool measurements used per assay. The interassay CV was calculated by taking the between-assay mean and standard deviation of a control sample (in this study a saliva pool consisting of four samples) included in all assays. The control samples from a saliva pool showed interassay CVs between 8.08 and 30.71%. The mean interassay CV of the 12 RIAs was 16.50%. Again, interassay CVs below 10% are considered as good and, as aforementioned, various factors could have produced this outcome. No cortisol samples were dismissed due to lower RIA quality, as there was no clear evidence of irregularities in the work process and as the overall RIA quality was in accordance with general RIA standards.

### Covariates

In accordance with previous research (e.g., Schultheiss et al., 2012; Stawski et al., 2013) a screening questionnaire was distributed that covered key factors which may affect salivary cortisol levels. Covariates included in this study were gender, age, education, job tenure, BMI, smoking status, exercise status, and time of wakeup. Smoking and exercise status were dichotomous variables that assessed if participants smoked or not and did endurance sports or not (in the following called smoking and exercise). At last, time of wakeup was included as an explaining variable as well.

## Results

**Figure 1:**
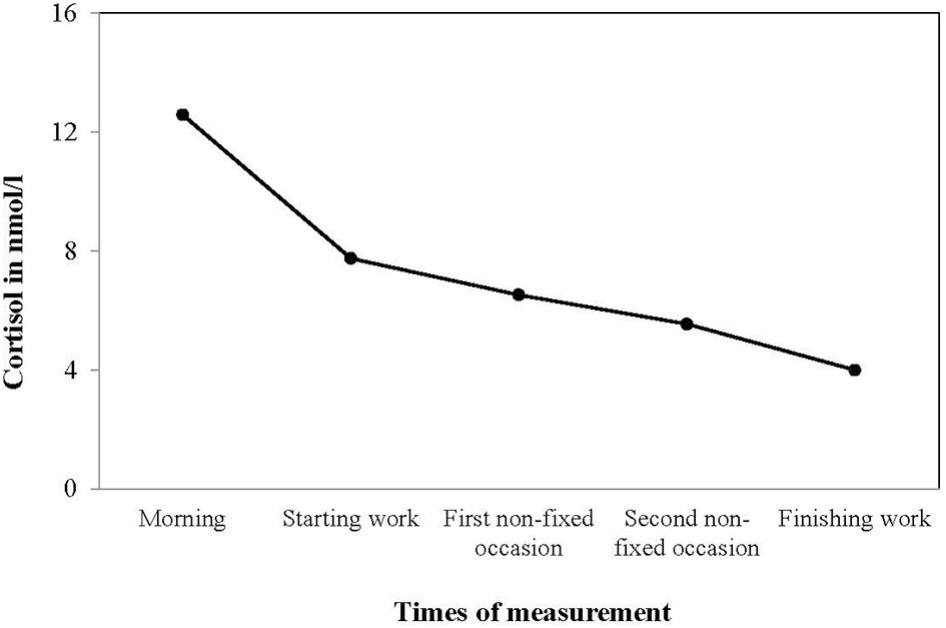
**Mean cortisol values (nmol/1, non-transformed) at five daily measurement occasions, reflecting a typical diurnal rhythm of cortisol secretion**.

For descriptive statistics of cortisol, non-log transformed values in nmol/l were used. In Figure 1, mean cortisol values of all five points of measurement are displayed, reflecting a typical diurnal rhythm of cortisol secretion with a peak in the morning hours and a gradual decline over the course of the work day. Descriptive statistics of collection times, mean cortisol levels and AUC are reported in Table 1. Descriptive statistics of day-level variables are shown in Table 2.

**Table 2:** **Zero-order correlations between day-level variables**.

**Variable**	**1**	**2**	**3**	**4**	**5**	**6**
1 Negative work events	–	0.09	0.25^**^	0.18^**^	−0.01	0.00
2 Time of waking		–	0.05	0.04	−0.36^**^	−0.13
3 Emotional exhaustion			–	0.36^**^	0.03	0.22^*^
4 Negative affect				–	0.10	0.07
5 Cortisol after finishing work					–	0.37^**^
6 AUC						–

*AUC, Total diurnal cortisol output*.

* *p < 0.05*,

** *p < 0.01*.

Before testing our hypotheses, it was necessary to establish that a multilevel data analytic approach was appropriate. We calculated intraclass correlation coefficients (ICCs) to determine the proportion of the variance in outcome variables that was attributable to within-person variability. Results suggest substantial intra-individual fluctuations in AUC (1 − ICC = 0.4095), cortisol levels after finishing work (1 − ICC = 0.6305), negative affect (1 − ICC = 0.5723), and emotional exhaustion after finishing work (1 − ICC = 0.5449), supporting the use of HLM.

### NWEs and total diurnal cortisol output

As indicated by estimates of regression coefficients displayed in Table 3, neither NWEs, γ = 0.204, *SE* = 0.53, *t*_(85)_ = 0.39, *p* = 0.701, nor time of waking, γ = 0.187, *SE* = 0.15, *t*_(84)_ = 1.28, *p* = 0.204, exhibited a significant association with AUC. Thus, we found no support for Hypothesis 1.

**Table 3:** **Multilevel estimates for models predicting total diurnal cortisol output (AUC)**.

**Variable**	**Null model**		**Model 1**		**Model 2**		**Model 3**	
	**Coefficient**	***t***	**Coefficient**	***t***	**Coefficient**	***t***	**Coefficient**	***t***
Intercept	17.134 (0.54)	31.75^***^	17.064 (0.52)	32.95^***^	17.064 (0.52)	32.95^***^	17.063 (0.52)	32.96^***^
Age			0.049 (0.06)	0.76	0.049 (0.06)	0.76	0.049 (0.06)	0.76
Gender^a^			−0.575 (1.06)	−0.54	−0.575 (1.06)	−0.54	−0.575 (1.06)	−0.54
Job tenure			0.041 (0.08)	0.50	0.041 (0.08)	0.50	0.041 (0.08)	0.50
Education^b^			1.222 (0.69)	1.78	1.222 (0.69)	1.78	1.220 (0.69)	1.78
Smoking^c^			0.045 (1.25)	0.04	0.045 (1.25)	0.04	0.046 (1.25)	0.04
Exercise^c^			0.538 (1.25)	0.43	0.538 (1.25)	0.43	0.539 (1.25)	0.43
BMI			−0.016 (0.13)	−0.11	−0.016 (0.14)	−0.11	−0.016 (0.14)	−0.12
Negative work events					0.204 (0.53)	0.39	0.163 (0.52)	0.31
Time of waking							0.187 (0.15)	1.28
−2 log likelihood (FIML)	842.51		835.45		835.30		833.68	
Δ−2 log likelihood			7.06		0.15		1.62	
Number of estimated parameter	3		10		11		12	

*The number of daily observations was 148, nested within 62 individuals. Coefficients are unstandardized estimates of regression coefficients. Standard errors appear in parentheses. FIML, full information maximum likelihood estimation*.

a *Gender is coded as 0, male; 1, female*.

b *Education is coded as 1, no formal education; 2, professional training; 3, higher professional training; 4, university degree*.

c *Smoking and exercise are coded as 0, no; 1, yes*.

*** *p < 0.001*.

### NWEs and cortisol levels after finishing work

There was no significant relationship between daily NWEs and cortisol levels after finishing work, γ = −0.022, *SE* = 0.07, *t*_(116)_ = −0.31, *p* = 0.756, after controlling for gender, age, education, tenure, BMI, exercise, and smoking. Time of waking, entered in Model 3 (see Table 4), significantly predicted cortisol levels after finishing work, γ = −0.045, *SE* = 0.02, *t*_(98)_ = −2.44, *p* = 0.017. In sum, Hypothesis 2 was not supported.

**Table 4:** **Multilevel estimates for models predicting daily cortisol levels right after finishing work**.

**Variable**	**Null model**		**Model 1**		**Model 2**		**Model 3**	
	**Coefficient**	***t***	**Coefficient**	***t***	**Coefficient**	***t***	**Coefficient**	***t***
Intercept	1.127 (0.06)	19.18^***^	1.136 (0.06)	19.91^***^	1.136 (0.06)	19.9^***^	1.102 (0.06)	19.13^***^
Age			−0.009 (0.01)	−1.26	−0.009 (0.01)	−1.26	−0.010 (0.01)	−1.40
Gender^a^			−0.040 (0.12)	−0.34	−0.040 (0.12)	−0.34	−0.003 (0.12)	−0.02
Job tenure			0.010 (0.01)	1.08	0.010 (0.01)	1.08	0.016 (0.01)	1.73
Education^b^			0.131 (0.08)	1.70	0.131 (0.08)	1.70	0.145 (0.08)	1.91
Smoking^c^			0.174 (0.14)	1.28	0.174 (0.14)	1.28	0.268 (0.14)	1.93
Exercise^c^			−0.003 (0.14)	−0.03	−0.003 (0.14)	−0.03	0.065 (0.14)	0.48
BMI			0.013 (0.01)	0.88	0.013 (0.01)	0.88	0.028 (0.02)	1.79
Negative work events					−0.022 (0.07)	−0.31	0.063 (0.07)	0.85
Time of waking							−0.045 (0.02)	−2.44^*^
−2 log likelihood (FIML)	351.55		346.63		346.53		269.45	
Δ−2 log likelihood			4.92		0.10		77.08^***^	
Number of estimated parameter	3		10		11		12	

*The number of daily observations ranged from 167 to 192, nested within 67 to 75 individuals. Coefficients are unstandardized estimates of regression coefficients. Standard errors appear in parentheses. FIML, full information maximum likelihood estimation*.

a *Gender is coded as 0, male; 1, female*.

b *Education is coded as 1, no formal education; 2, professional training; 3, higher professional training; 4, university degree*.

c *Smoking and exercise are coded as 0, no; 1, yes*.

* *p < 0.05*,

*** *p < 0.001*.

### NWEs and negative affect after finishing work

There was a significant positive relationship between NWEs and negative affect after finishing work, γ = 0.159, *SE* = 0.05, *t*_(82)_ = 2.94, *p* = 0.004, supporting Hypothesis 3 (see Table 5).

**Table 5:** **Multilevel estimates for models predicting daily negative affect right after finishing work**.

**Variable**	**Null model**		**Model 1**		**Model 2**	
	**Coefficient**	***t***	**Coefficient**	***t***	**Coefficient**	***t***
Intercept	1.394 (0.05)	27.85^***^	1.396 (0.05)	28.29^***^	1.396 (0.05)	28.15^***^
Age			0.003 (0.01)	0.52	0.004 (0.01)	0.69
Gender^a^			−0.123 (0.10)	−1.20	−0.053 (0.10)	−0.54
Job tenure			−0.007 (0.01)	−0.93	−0.008 (0.01)	−1.07
Education^b^			0.000 (0.06)	0.00	0.003 (0.06)	0.06
Negative work events					0.159 (0.05)	2.94^**^
−2 log likelihood (FIML)	364.29		361.70		341.60	
Δ−2 log likelihood			2.59		10.00^**^	
Number of estimasted parameters	3		7		10	

*The number of daily observations ranged from 196 to 234, nested within 72–83 individuals. Coefficients are unstandardized estimates of regression coefficients. Standard errors appear in parentheses. FIML, full information maximum likelihood estimation*.

a *Gender is coded as 0, male; 1, female*.

b *Education is coded as 1, no formal education; 2, professional training; 3, higher professional training; 4, university degree*.

** *p < 0.01*,

*** *p < 0.001*.

### NWEs and emotional exhaustion after finishing work

Adding NWEs (see Model 2, Table 6) significantly improved model fit, Δ^−2^ log likelihood = 13.76, $\chi_{\left(4\right)}^{2}$ = 4.36, *p* < 0.001. In line with this, NWEs positively predicted emotional exhaustion after finishing work, γ = 0.169, *SE* = 0.05, *t*_(122)_ = 3.45, *p* < 0.001, consistent with Hypothesis 4.

**Table 6:** **Multilevel estimates for models predicting daily emotional exhaustion right after finishing work**.

**Variable**	**Null model**		**Model 1**		**Model 2**	
	**Coefficient**	***t***	**Coefficient**	***t***	**Coefficient**	***t***
Intercept	2.218 (0.06)	39.09^***^	2.223 (0.05)	40.56^***^	2.224 (0.06)	40.29^***^
Age			0.008 (0.01)	1.21	0.008 (0.01)	1.16
Gender^a^			0.052 (0.11)	0.46	0.054 (0.11)	0.47
Job tenure			−0.016 (0.01)	−1.77	−0.015 (0.01)	−1.72
Education^b^			0.038 (0.07)	0.55	0.036 (0.07)	0.53
Negative work events					0.169 (0.05)	3.45^***^
−2 log likelihood (FIML)	315.00		310.64		296.88	
Δ−2 log likelihood			4.36		13.76^***^	
Number of estimated parameters	3		7		8	

*The number of daily observations ranged from 195 to 196 nested within 72 individuals. Coefficients are unstandardized estimates of regression coefficients. Standard errors appear in parentheses. FIML, full information maximum likelihood estimation*.

a *Gender is coded as 0, male; 1, female*.

b *Education is coded as 1, no formal education; 2, professional training; 3, higher professional training; 4, university degree*.

*** *p < 0.001*.

### *Post-hoc* analyses

Building on Dickerson's and Kemeny's meta-analysis (2004) showing the largest effects sizes (*d* = 0.67) in laboratory studies for tasks with social-evaluative threats, we further analyzed whether only NWEs that can be characterized as socially threatening events (e.g., negative interactions with one's supervisor) show significant associations with cortisol activity. In our study, after reporting NWEs, participants answered dichotomous questions regarding the specific type of stressor. Specifically, they indicated whether the reported NWE involved a problem with a colleague, supervisor, patient, and/or lack of support from their supervisor/s (0 = *no*, 1 = yes). We examined whether across the five measurement occasions there were significant within-person associations between socially threatening events and AUC and cortisol levels after finishing work, respectively. In addition, we analyzed whether the total number of socially threatening events was associated with AUC and cortisol levels after finishing work, respectively. Contrary to Dickerson and Kemeny (2004), we did not find any significant associations between socially threatening events and cortisol activity. Detailed results may be obtained from the first author.

## Discussion

The goal of the present diary field study was to gain insight into the effects of NWEs on employees' physiological and psychological distress responses. Field research on physiological reactions to occupational stressors is scant and has generated inconsistent findings. In addition, the simultaneous examination of employees' physiological and psychological responses to daily NWEs has been neglected in previous research. Clearly, an integration of both employees' physiological reactions and their self-reported psychological distress is central to advancing our understanding of the detrimental effects of taxing situational demands in the workplace on employees' distress reactions. In light of this, we investigated the effects of daily NWEs on employees' daily physiological (i.e., total diurnal cortisol output and cortisol levels toward the end of the work day) and psychological (i.e., negative affect and emotional exhaustion toward the end of the work day) distress. As opposed to prior research, we pursued a new methodological approach by linking salivary sampling and data collection on psychological well-being directly to the reported stressful work events in order to capture immediate reactions to the NWEs.

Contrary to our assumptions regarding employees' physiological reactions to NWEs, we did not find any significant associations between the number of NWEs and employees' cortisol levels. In line with previous research (for reviews, see Sonnentag, 2015; Ilies et al., 2016), we were, however, able to provide support for our assumptions regarding daily *psychological* stress reactions to NWEs: On days with higher numbers of NWEs employees reported higher levels of negative affect and emotional exhaustion toward the end of the work day.

Given that salivary cortisol and perceived stress emerge as unrelated in many studies, Hellhammer et al. (2009) argue that this finding is due to the complexity of the path from perceived stress to physiological processes. The inclusion of person- and time-specific factors might be necessary in order to better understand the observed non-significance of relationships between absolute cortisol levels and cognitive or subjective distress indicators.

### Theoretical and practical implications

Our study acknowledges earlier calls of researchers to take account of human physiology in organizational research (Heaphy and Dutton, 2008) and to measure psychosocial work stressors by means of EMA (Beal and Weiss, 2003, as outlined by Ilies et al., 2016). Our multilevel results on short-term fluctuations of NWEs and within-person effects of NWEs on daily psychologcial but not endocrine stress indicators help to inform theory about the complex interplay of different types of primary AL processes.

Our finding that daily NWEs are only associated with psychological but not with physiological distress merits closer scrutiny. It might be that other physiological distress indicators, such as blood pressure, are more strongly affected by NWEs (Ilies et al., 2010a,b). Interestingly, Ilies et al. (2010a) found very low within-person correlations (0.08–0.22) between blood pressure and psychological distress reactions (i.e., burnout and strain), resembling our very low within-person correlations (0.03–0.22) between cortisol and psychological distress reactions (i.e., negative affect and emotional exhaustion). Ilies et al. (2016) concluded that physiological stress indicators are poor correlates of psychological distress reactions and that AL processes may differ at the within- and between-person level of analysis. The consequences of coherence and dissociation of human response systems (experiental, behavioral, and physiological) have been of research interest in other psychology disciplines (e.g., Evers et al., 2014). Further consideration of the conditions and consequences of coherence and dissociation of different response systems in the work context would inform our knowledge about how work stressors impact employees' health and well-being.

Daily social-evaluative events (e.g., negative interactions with one's supervisor) were not associated with employees' salivary cortisol levels, inconsistent with prior findings (Dickerson and Kemeny, 2004). However, our study was conducted as an EMA, thus implying a dynamic intraindividual level of analysis. Future research should replicate our findings and shed more light on the type of NWE in order to provide more information on, for instance, the duration, frequency, and intensity of the stressor.

In our study, within individuals, NWEs were associated with higher daily negative affect and daily emotional exhaustion after the workday. As the primary, secondary, and tertiary processes of the AL model (McEwen, 1998) interact with each other, secondary and tertiary AL processes (e.g., cholesterol problems, depression) are likely to occur. Organizations should be aware of the detrimental impact of occupational stressors on employees' psychological well-being. For example, conflict management trainings and job design analysis might help to reduce workplace stressors.

### Strengths, limitations, and avenues for future research

A strength of the present research is the combination of an event-sampling approach with both physiological and psychological distress reaction measures. This procedure allowed us to link physiological and psychological distress reactions to NWEs in a naturalistic context. By measuring salivary cortisol at five independent measurement occasions across each work day, we were able to depict employees' diurnal pattern of cortisol secretion. Collection, storage, preparation, and analysis of cortisol data was conducted with utmost caution to ensure valid interpretation of the endocrinological information obtained on a daily basis. Future studies might build on this study and extend our findings by including secondary and tertiary AL processes (cf. Ilies et al., 2016).

Regarding limitations, our sample consisted of healthcare professionals. Findings need to be replicated with random samples in more diverse occupational settings. Second, we collected salivary cortisol 3–5 times per day. More frequent samples of daily cortisol may provide more fine-grained data to detect links between daily stressors and cortisol secretion. Third, diary studies including repeated salivary cortisol collection are quite demanding. Participants might have felt obliged to report an event at the two fixed occasions, but due to time constraints, we might have missed more stressful events at other times. Finally, we focused on NWEs, neglecting interactions between positive and NWEs (Bledow et al., 2011).

It is possible that our approach of mixing time- and event-contingent event-sampling might have biased the data. For example, when participants felt obliged to report an event and at other times omitted a NWE due to time constraints, a bias in different directions could occur. In order to increase representativeness of the daily events, future studies could combine fixed time points for measuring cortisol with random time points during the day to assess NWE. As for the representativeness of the cortisol data, cortisol information used to calculate the AUC may refer to different origins on different days. One option to handle this limitation would be to collect additional data as control measurement for comparisons within-person. Participants could provide saliva samples at the same time as each non-fixed NWE on a day when the NWE is not experienced. On this basis, cortisol levels following NWE could be compared to the participant's typical cortisol level at that specific time of day.

## Conclusion

In this study, daily within-person effects of NWEs on both employees' physiological (i.e., salivary cortisol levels) and psychological (i.e., negative affect and emotional exhaustion) distress were examined. Findings support associations of NWEs with self-reported psychological but not physiological distress levels. Our study broadens the scope of occupational stress research by simultaneously investigating physiological and psychological stress reactions to NWEs on a short-term within-person level. We hope that our findings stimulate further research integrating neuroendocrinological and psychological stress indicators to contribute to the literature on (day-level) determinants of distress responses in the workplace, thereby paving the way for the development of efficient intervention programs designed to sustain employee well-being.

## Author contributions

Both authors have been involved in the planning, data collection, data analysis, and writing stages of the manuscript.

## Funding

This research was funded by a grant from the Staedtler Foundation (39/10), which is gratefully acknowledged.

### Conflict of interest statement

The authors declare that the research was conducted in the absence of any commercial or financial relationships that could be construed as a potential conflict of interest.
